# The Development and Characterisation of a P(3HB-*co*-4HB)–Bioactive Glass–Graphene Hydrogel as a Potential Formulation for Biomedical and Therapeutical Translation

**DOI:** 10.3390/gels10010085

**Published:** 2024-01-22

**Authors:** Nik S. A. N. Sharifulden, Lady V. Barrios Silva, Sean P. Nair, Amirul A. A. Abdullah, Siti N. F. M. Noor, Michael Sulu, Linh T. B. Nguyen, David Y. S. Chau

**Affiliations:** 1 Division of Biomaterials and Tissue Engineering, UCL Eastman Dental Institute, University College London, Royal Free Hospital, Rowland Hill Street, London NW3 2PF, UK; nik.aliaa.19@ucl.ac.uk (N.S.A.N.S.); lady.silva.17@ucl.ac.uk (L.V.B.S.);; 2 Department of Microbial Diseases, UCL Eastman Dental Institute, University College London, Royal Free Hospital, Rowland Hill Street, London NW3 2PF, UK; sean.nair@ucl.ac.uk; 3 School of Biological Sciences, Universiti Sains Malaysia, Gelugor 11800, Malaysia; 4 Advanced Medical and Dental Institute, Universiti Sains Malaysia, Bertam, Kepala Batas 13200, Malaysia; fazliah@usm.my; 5 Department of Biochemical Engineering, University College London, Bernard Katz Building, London WC1E 6BT, UK

**Keywords:** biomaterials, polyhydroxyalkanoate, skin, wound healing, tissue engineering, cell culture, (microbial) fermentation, dermal, biopolymer

## Abstract

The clinical management of wounds is known to be a significant challenge: not only does the dressing need to ensure and provide the appropriate barrier and healing characteristics, but consideration of patient compliance concerning comfort, functionality, and practicality also needs to be included. The poly(3-hydroxybutyrate-*co*-4-hydroxubutyrate) (P(3HB-*co*-4HB)) copolymer, isolated from *Cupriavidus malaysiensis* USM1020 (*C. malaysiensis* USM1020), was produced in the presence of excess carbon sources (1,4-butanediol and 1,6-hexanediol) using either a shake flask cultivation process or a bioreactor fermentation system. P(3HB-*co*-4HB) is widely known to be biodegradable and highly biocompatible and contains a tuneable 4HB monomer molar fraction, which is known to affect the final physicochemical properties of the intracellular copolymer. In this paper, we describe not only the fabrication of the polymeric gel but also its optimised profiling using a range of physical and mechanical techniques, i.e., SEM, FTIR, DMA, DSC, and WCA. The further enhancement of the gel through additional functionalisation with sol-gel-derived bioactive glass and liquid-exfoliated graphene was also investigated. The biocompatibility and biological characterisation of the substrates was assessed using murine osteoblasts (MC3T3), human primary dermal fibroblasts (HDFs), human fibroblast (BJ) cells, and standard cell culture assays (i.e., metabolic activity, LDH release, and live/dead staining). In short, P(3HB-*co*-4HB) was successfully isolated from the bacteria, with the defined physico-chemical profiles dependent on the culture substrate and culturing platform used. The additional enhancement of the copolymer with bioactive glass and/or graphene was also demonstrated by varying the combination loading of the materials, i.e., graphene resulted in an increase in tensile strength (~11 MPa) and the wettability increased following the incorporation of bioactive glass and 0.01 wt% graphene (WCA ~46.3°). No detrimental effects in terms of biocompatibility were noticed during the 7 days of culture in the primary and established cell lines. This study demonstrates the importance of optimising each of the individual components within the biocomposite and their relationship concerning the fine-tuning of the material’s properties, thus targeting and impacting the endpoint application.

## 1. Introduction

Skin, as the body’s largest organ, not only acts as a barrier to the outer environment but also actively protects and controls a number of bodily functions as well as enabling touch sensations. As such, any damage or trauma to the skin, if not treated appropriately, may lead to a range of complications, which, in extreme cases, can result in death. It has been reported that around 2 million people live with non-healing wounds in the United Kingdom, which can be categorised under acute or chronic wounds and usually as a result of burns, traumatic accidents, or diseases [1]. The dynamic process of wound healing starts with vasoconstriction during haemostasis, followed by an inflammatory response to remove damaged tissue and bacteria, before the formation of granulation and connective tissue during the proliferation stage, which is then ultimately remodelled as the wound matures. However, additional co-factors (e.g., age, lifestyle, and existing diseases) further intensify the repair and regeneration burden, and may even compound the damage due to possible re-infections, scarring tissue, increased (financial/time) costs, and extended rehabilitation duration. At present, there is an extensive range of wound dressings available that include traditional cotton gauze and certain specialised bio-based materials. However, limitations, such as frequent removal, low water retention, patient compliance, clinical procedure, and inconsistent supply, have driven the need to seek alternatives [2,3]. At present, the majority of research in the area concerns the formulation of novel (bio)materials, especially those that can be enhanced or manipulated with various functionalities to further elicit parameters such as cell viability, bioactivity, chemical reactivity, mechanical and physical characteristics, and reduced degradation rates, whilst maintaining the primary objective of barrier formation and healing [4,5].

**Figure 1:**
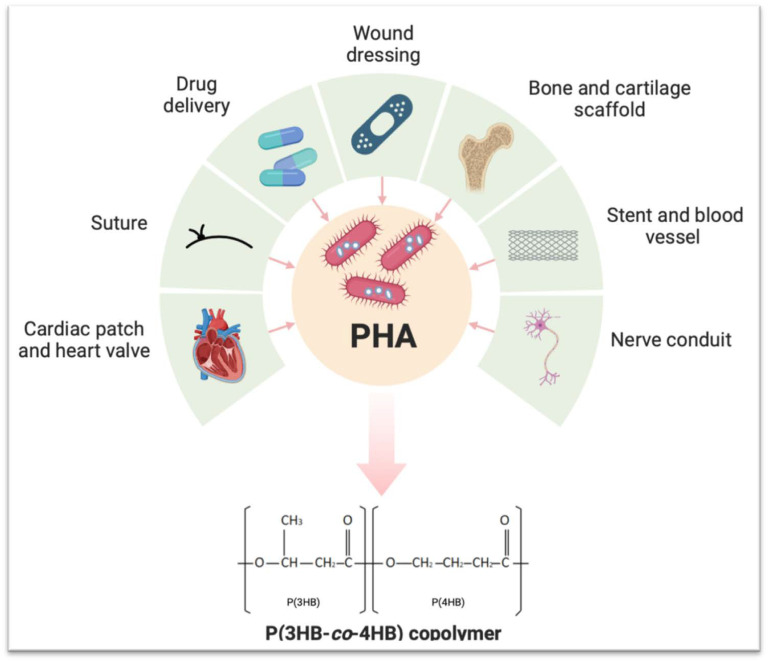
Several PHA-based biomedical engineering applications and one PHA intracellular bacterial versatile derivations, P(3HB-*co*-4HB) copolymer, resulted from excess carbon sources and an environment with limited nutrients.

In this paper, we report on the fabrication and development of the P(3HB-*co*-4HB) copolymer, a naturally derived material from a microorganism, in the development of a novel advanced hydrogel system. This substrate can be tailored and bioengineered from its precursors, which, in turn, can be manipulated to control their final characteristics and material profile. Moreover, the copolymer allows the integration of additional components and can be adapted to be a stimuli-responsive material. The poly(3-hydroxybutyrate-co-4-hydroxubutyrate), P(3HB-*co*-4HB), copolymer is a member of the super-family of polyhydroxyalkanoate (PHA) polyesters with both of the monomer components being native metabolites in the human body, hence its reported high biocompatibility [6,7]. Being microbial-derived, the use of such a copolymer is in line with the Sustainable Development Agenda adopted by the United Nations since 2015 (https://sdgs.un.org/goals, accessed on 8 August 2023) to encourage a higher production of biodegradable products from natural sources [8] as well as being an animal-free-derived material. Furthermore, the copolymer has highly tuneable physico-chemical characteristics, thus making it attractive for translational applications due to its customisability, which is related to the percentage of each monomer (Figure 1). The introduction of an additional copy of the polyhydroxyalkanoate synthesie gene (*phaC*) on a multicopy plasmid can increase the 4HB content, resulting in a more elastic copolymer that would be very much suitable as a wound dressing [9].

The additional enhancement and/or functionalisation of materials can overcome certain polymer matrix deficiencies, such as in hydrophilicity, mechanical behaviour, and morphological structure, which ultimately lead to a better biocompatibility and cellular interaction. To that end, bioactive glass and graphene were incorporated within the gel composite to improve the physicochemical properties of the P(3HB-*co*-4HB) copolymer. Bioactive glass is a third-generation biomaterial, with an extensive usage history in both hard and soft tissue regeneration and also drug delivery [10]. The material is known to promote vascularisation and modulate anti-inflammatory molecules and growth factors upon the dissolution of its ions, by stimulating the secretion of angiogenic growth factors, which in turn enhances the proliferation of microvascular endothelial cells and revascularisation for an efficient process of wound healing [11]. This study utilises bioactive glass with the “golden” ratio of 46.1 mol% SiO_2_, 24.4 mol% Na_2_O, 26.9 mol% CaO, and 2.6 mol% P_2_O_5_ using the sol-gel method, which results in a bioactive, highly resorbable product as compared to the traditional melt-derived bioactive glasses with a similar composition [12,13]. Moreover, the additional advantages of the sol-gel technique, such as higher specific surface area and purity with lower working temperatures during production, result in a significantly more economical and sustainable process [12,13]. Graphene, a two-dimensional carbon nanomaterial arranged in a honeycomb lattice, has been known to have desirable characteristics since its discovery in 2004 by Novoselov and Geim and can be easily prepared by the micromechanical exfoliation of graphite, better known as the ‘Scotch tape’ method [14]. Its properties include a high surface area, high Young’s modulus, and distinct thermal, electrical, and optical properties [15,16]. Graphene was prepared by the liquid exfoliation technique of graphite, a more reliable top-down approach to obtaining graphene that is not structurally defective and is of high quality and economically viable [14,15,17].

Hence, this study aims to improve the functionality of the P(3HB-*co*-4HB) copolymer by harnessing the potential of bioactive glass and graphene to elevate the physical and mechanical attributes of the composite. Finding the right balance between the optimal mechanical strength and wettability, which ultimately affects the degradation behaviour, presents a notable challenge in effectively transitioning biocomposites into functional wound dressings within the biomedical field. The established scientific range for hydrophobicity is characterised by static angles (θ) surpassing 90°, while angles below 90° typically indicate hydrophilicity, which are associated with potentially facilitating improvement in cell adhesion [18,19]. However, despite extensive efforts dedicated to designing and developing effective wound dressings, there is a persistent absence of dressings that satisfy all the necessary criteria for wound healing [11], including its necessity for a good range of tensile strength with a high modulus and flexibility, rendering a robust and durable composite, subsequently impacting its resorbable rate [20].

Taking the results together, this study highlights the potential exploitation of P(3HB-*co*-4HB) isolated from *C. malaysiensis* USM1020. This strain is known to possess a superb phaCAB operon (with additional copies of *phaC* gene), which contributes to the high production of the P(3HB-*co*-4HB) copolymer with a high 4HB monomer composition, depending on certain parameters, including the method of cultivation, and this study specifically compares the production of the hydrogel polymer using the shake flask and bioreactor methods [6,9]. Moreover, this study also discusses the importance of the assessment of the individual parameters of each component, i.e., the bioactive glass and/or graphene, which contribute to the several vital characteristics that ultimately affect the final working conditions of the combined composite for potentially suitable clinical and therapeutical applications.

## 2. Results and Discussion

### 2.1. Optimisation and Characterisation of P(3HB-co-4HB) Copolymer Biosynthesis

P(3HB-*co*-4HB) is a microbial-derived copolymer, the properties and final characteristics of which are highly dependent on the composition and structure of the bioengineered polymer chain. The culture of *C. malaysiensis* USM1020 was achieved using both the shake flask and stirred tank bioreactor fermentation techniques. The bacterial growth (i.e., biomass) from each of the fermentation processes was monitored using optical density measurements. The average of the optical density measurements from three different trials of each fermentation process is shown in Table 1. For ease of comparison for the final weight of the recovered crude copolymer, the same amount of the initial weight of the lyophilised cells was set at 1.25 g. It can be seen in Table 1 that only slightly lower yields of the copolymer were obtained using the shake flask culture (51.72%) compared to the fermentation in a bioreactor (55.19%), which was opposed to our expectations of observing a much significantly higher accumulation of biomass production using the latter fermentation process due to well-controlled conditions during growth. However, our results showed a comparably lower value of biomass produced and 4HB monomer composition. Norhafini et al. reported in their study that using the same strain of *C. malaysiensis* USM1020 through fermentation in a bioreactor was able to produce a higher percentage of PHA content around 75 wt% and PHA concentration of 20.3 g/L, with a 4HB monomer composition of 95 mol%, when cultured with similar parameters in comparison to our study [6.] This is associated with its genes containing an extra copy of the phaCAB operon (with additional copies of the *phaC* gene) within the plasmid [6,8,9], although our study obtained a low production yield from the bioreactor conditions. 

**Figure 2:**
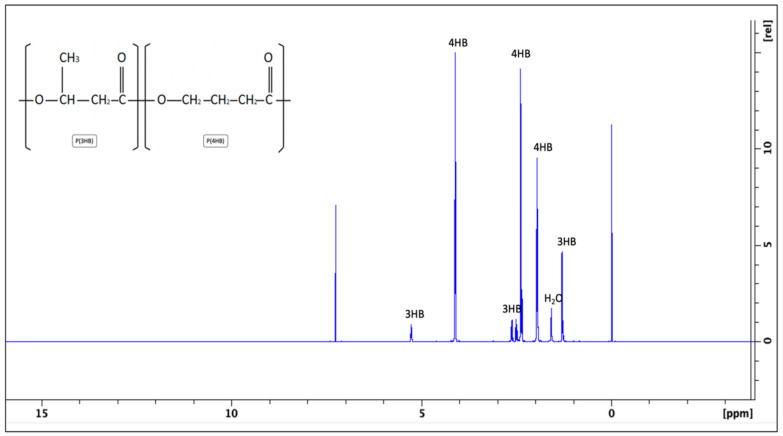
^1^H-NMR shows the comparative composition results based on a higher percentage of 4HB monomers in the purified crude copolymer, which correlates to the ability of the composite casting and characterisations.

The recovered crude copolymer from the shake flask fermentation was then analysed using ^1^H-NMR to study the presence of both the 3HB and 4HB monomer content using the typical ^1^H-NMR spectrum, yielded as shown in Figure 2. The several dominant peaks at 2 ppm, 2.4 ppm, and 4 ppm correspond to 4HB monomers; meanwhile, 3HB monomers were noted at several peaks, at 1.3 ppm, 2.5 ppm, 2.6 ppm, and 5.2 ppm. Each of these peaks represents the number of hydrogen atoms in each of the monomers.

The crude copolymer from the shake flask fermentation, due to the unavoidable external environment, was further divided into batch A and batch B; the condition of the strain was suspected to be compromised in the latter batch, as depicted in Table 2. The consistency and reproducibility of each batch were based on at least two times of ^1^H-NMR testing. Integration between the dominant relative peaks of 4HB monomers compared to the 3HB monomers was then calculated and yielded a ratio of adjacent protons between these two monomers, where they were represented by the percentage of monomer composition. Batch A had a higher accumulation of the 4HB monomer, around 84–89% compared to batch B, at around 68–69%. The variations are further explained in the next section, as they affect the feasibility of each batch in making the composites.

Both the composition and percentage of each monomer present in the copolymer backbone, as confirmed in Figure 2, had an effect on the properties of the final copolymer. The P(3HB-*co*-4HB) copolymer with a high 4HB monomer percentage can be bioengineered and tailored using a bacterium with appropriate precursors, as the bacteria naturally produce the copolymer for intracellular energy storage. The presence and percentage of both monomers highly depend on the conditions supplied during growth, i.e., the types of carbon sources, ratio of carbon to nitrogen, and method of production [21]. Huong et al. reported a pilot study of scaling up the production of PHA by controlling and optimising parameters during fermentation, including agitation speed and oxygen supply, which increased the PHA content from 28 wt% to 63 wt% with a reduction in other bacterial biomass. This had the effect of reducing the molecular weight and mechanical strength of the copolymer when a lower agitation and aeration rate were employed, which improved the resulting copolymer suitably for its intended application.

### 2.2. Composite Casting of the Copolymer

**Figure 3:**
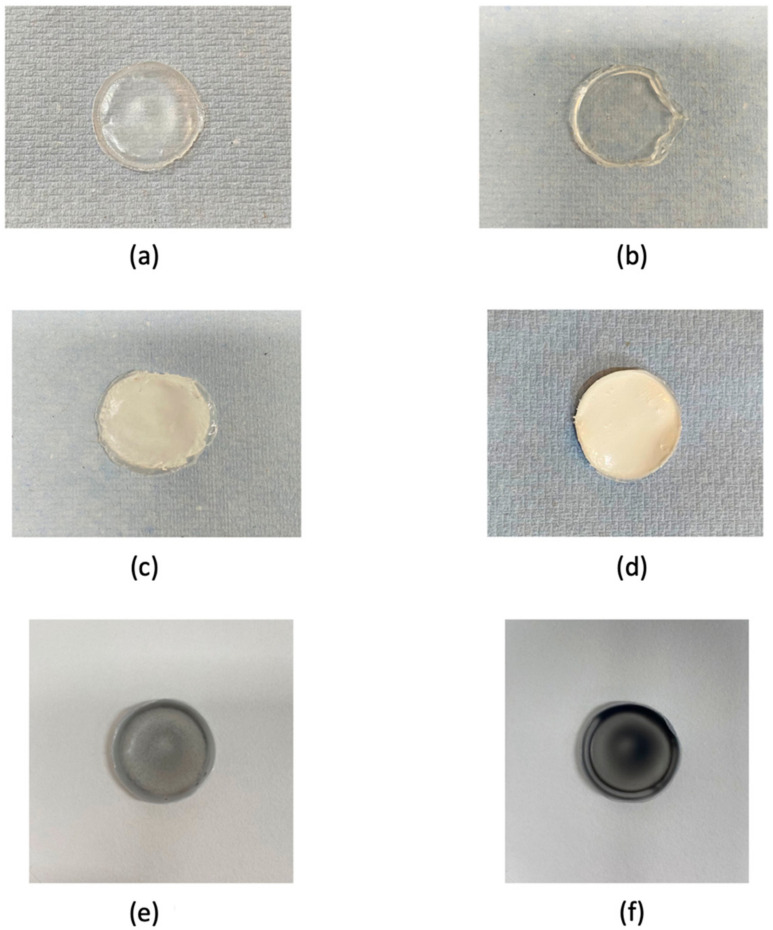
Optical observation of P(3HB-*co*-4HB) based the composite cast from (**a**) batch A (PHA 89% 4HB), (**b**) batch B (PHA 68% 4HB), (**c**) PHA 68% 4HB cast with 1 wt% bioactive glass (PHA 68% 4HB-Bioactive glass), (**d**) PHA 68% 4HB cast with 5 wt% bioactive glass, (**e**) PHA 89% 4HB-Bioactive glass—0.01% Graphene, and (**f**) PHA 89% 4HB-Bioactive glass—0.1% Graphene.

Following the determination of the monomer content of the composite using NMR, the copolymer and crude copolymer from both batch A and batch B were subjected to composite casting using the solvent casting method, as visually observed in Figure 3a,b, from this point onwards called PHA 89% 4HB and PHA 68% 4HB, respectively. Differences in the opacity were clearly seen, as this affected not only the visualisation of the copolymer but also its processing capability where a lower 4HB (batch B; PHA 68% 4HB) in the copolymer led to a more viscous and gel-like structure, which was potentially due to the presence of more ester carbonyl bonds in the batch B copolymer backbone, which is further discussed in the FTIR results. This led to a further hindrance in incorporating bioactive glass and graphene, which were utilised in this study to improve the surface functionalisation of the copolymer. 

An attempt to improve the fabrication feasibility for the batch B (PHA 68% 4HB) crude copolymer was conducted by incorporating the sol-gel-derived bioactive glass at a different percentage. This was also conducted to assess the batch-to-batch variation after the incorporation of the sol-gel-derived bioactive glass, which resulted in an evenly distributed bioactive glass in the PHA 68% 4HB copolymer composites. A thicker and more opaque composite was obtained after the incorporation of 1 wt% bioactive glass, as seen in Figure 3c, from this point onwards called PHA 68% 4HB-Bioactive glass. However, upon increasing the concentration of bioactive glass (5 wt%), the thickness and opacity were further increased, as shown in Figure 3d, together with the high levels of cytotoxicity that make it unfeasible for further use, which is discussed further in later sections.

Figure 3e,f show composites cast using the batch A (PHA 89% 4HB) crude copolymer with the optimised 1 wt% bioactive glass and 0.01 wt% and 0.1 wt% of the liquid-exfoliated graphene, from this point onwards called PHA 89% 4HB-Bioactive glass—0.01% Graphene and PHA 89% 4HB-Bioactive glass—0.1% Graphene, respectively. There was an increase in opacity as the concentration of graphene increased.

A study by Faisalina et al., which reported the utilisation of several molar fractions of P(3HB-*co*-4HB) optimised at 70 mol%, provided the desired physicochemical properties, including particle size distribution and also efficient drug encapsulation and release, encapsulating the anticancer drug docetaxel [2]. This study suggested that the variations in physical and thermal characteristics were highly dependent on the monomeric composition and properties, especially the percentage of the 4HB monomer, which influenced the elasticity and crystallinity range of the copolymer [22]. It suggested that a higher percentage of 4HB monomers lowered the crystallinity of the copolymer, which makes it more malleable and suitable for the incorporation of other materials. Specifically in our study, brittle materials, like bioactive glass and the monolayer of liquid-exfoliated graphene, were incorporated while retaining the copolymer form and can be suitably applied as wound dressings. Hence, in our study, the high percentage of 4HB monomer of 89% yielded a more stable composite than 68% of 4HB monomers, which was able to accommodate the sol-gel-derived bioactive glass and liquid-exfoliated graphene better; it was then further optimised and studied for its chemical, physical, and structural properties.

### 2.3. ATR-FTIR

**Figure 4:**
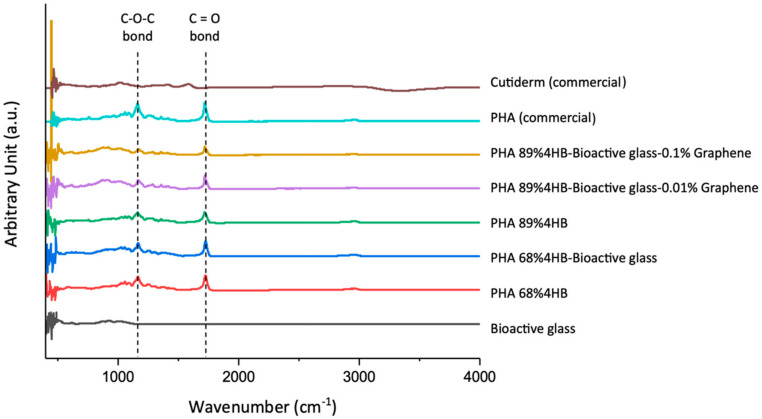
ATR-FTIR showed the presence of vital functional groups in all the cast composites.

Figure 4 summarises the ATR-FTIR spectra for all composites, including the bioactive glass in pure form. Pure graphene was not included due to being in liquid form and not suitable for this test. The confirmation of the chemical composition and functional groups was important in monitoring the chemical modifications and homogeneity of each compound in the composites. The presence of a characteristic absorption band at 1717 cm^−1^ was associated with the presence of short-chain-length PHAs (scl-PHAs), including P(3HB-*co*-4HB), mainly due to the C=O stretching vibration of the ester carbonyl of the crystalline carbonyl groups, similar to the results of other published studies, where this major ester carbonyl stretch band of the PHA was within the range of 1715–1740 cm^−1^ [23,24,25]. Of note is that PHA 68% 4HB showed a slightly more intense and sharper peak at this band compared to PHA 89% 4HB, and may correspond to the increased viscosity. It has been reported in other papers that this particular band is indicative of increased hydrogen bonding and ionic interactions with oxygen atoms acting as hydrogen bond acceptors and that this affects the viscosity of the composite [26]. The amorphous band that signifies the asymmetric stretching vibration of the C-O-C group can also be seen around 1180 cm^−1^. Of note, the intensity of these bands was similar between PHA 68% 4HB and PHA 68% 4HB-Bioactive glass. However, even lower intensity bands can be clearly seen for the PHA 89% 4HB incorporated with bioactive glass and increments in graphene concentrations, especially at 0.1% graphene. There was also the appearance of bands at wavenumber values between 1000 and 1450 cm^−1^ that indicate the presence of methyl CH_3_, methylene CH_2_ deformations, and C-O stretches of PHA-based composites. 

The presence of broad bands for bioactive glass powders at 1039 cm^−1^ and 930 cm^−1^ indicates the presence of the Si-O-Si stretching of non-bridging oxygen atoms, and the Si-O-Si asymmetric stretching of bridging oxygen atoms in the silicate tetrahedron can be seen with a higher and broader intensity around these bands for the composites compared to the ones without this material [27].

Other studies have highlighted the sensitivity of some IR bands to the degree of crystallinity from around 1000 to 1450 cm^−1^, as found in our study, where the intensity of these bands became broader with the incorporation of bioactive glass and lowered as the percentage of graphene increased [23]. The broader bands after the incorporation of bioactive glass were mainly due to the presence of the Si-O-Si stretching of non-bridging oxygen atoms and the Si-O-Si asymmetric stretching of bridging oxygen atoms within the silicate tetrahedron [28].

As for the commercial samples, similar peaks were seen for PHA-based ones, and a narrow OH stretching vibration was seen for Cutiderm due to the decrease in hydrogen bonds between the hydroxyl functional groups and the formation of a chelating structure of hydroxyl and carboxylate groups of alginate with calcium ions [29].

### 2.4. DSC

**Figure 5:**
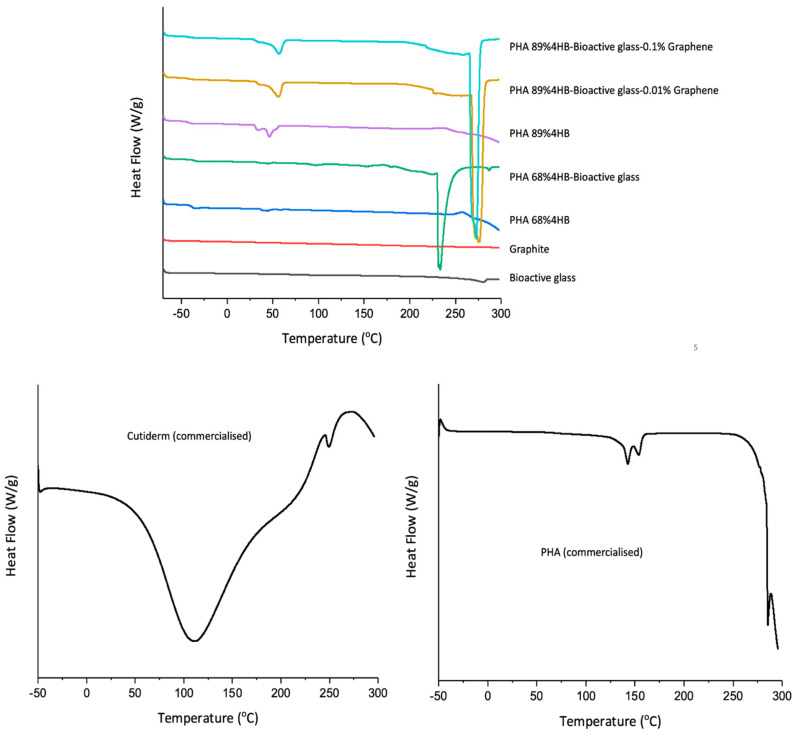
DSC thermograms showing that the low melting temperature exhibited by the PHA-based composites after the incorporation of bioactive glass and graphene was maintained.

Meanwhile, differential scanning calorimetry (DSC) was employed to study the thermal profile of the composites. Both the PHA 68% 4HB and PHA 89% 4HB copolymers showed a slightly different heat pattern, where PHA 89% 4HB had clear dual melting peaks at around 50 °C compared to PHA 68% 4HB. It is mostly due to a higher 4HB percentage within the copolymer chain that affects its functional mobility. This also corresponds to a lower second melting peak of PHA 68% 4HB at around 230 °C after the incorporation of bioactive glass, compared to PHA 89% 4HB, where after the incorporation of bioactive glass and graphene, the melting peak shifted to a higher temperature at around 275 °C, as seen in Figure 5.

Also of note, the melting temperature of all the PHA-based composites, even after the incorporation of bioactive glass and graphene, was maintained at ~50 °C. This is crucial as it signifies that the incorporation of both materials, especially graphene, at percentages of 0.01% and also 0.1% had no detrimental impact on the heat flow, although the dual peak seen at 50 °C was smoothed out after the incorporation of both bioactive glass and graphene, compared to the PHA 89% 4HB copolymer.

Previous studies showed a thermal profile for a similar P(3HB-*co*-4HB) copolymer derived from the same bacterial strain as that used in this study with a higher melting temperature of 62 °C [6,7], whereas our study observed a lower melting temperature of around 50 °C. The lower melting temperature allows the stable melting and modest loss of the molecular mass of the composite, which indicates that our study achieved a copolymer with a better 4HB percentage to provide a broader processing window [6,30]. Both PHA 89% 4HB and PHA 68% 4HB exhibited dual melting peaks, which suggests there was a recrystallisation process within the copolymer, although this gradually became smoother, especially for the first endothermic peak around 40 °C after the incorporation of bioactive glass, mainly caused by water molecules and residual inorganic precursors [13,31]. Interestingly, the melting temperature of the copolymer was maintained even after the incorporation of bioactive glass and graphene, especially with PHA 89% 4HB, suggesting the stability of the copolymer that was the carrier for the composite. A higher crystalline peak temperature was observed across all copolymers at around 235 °C for PHA 68% 4HB and 275 °C for PHA 89% 4HB. The shift in the crystallisation peak temperature, which increased after the incorporation of graphene, suggests the role of graphene in increasing the thermal crystallisation of the composite and could potentially control the rate of degradation of the composites. A study by Santos et al. also showed a similar change in crystallisation behaviour with incremental concentrations of graphene nanoplatelets within a polyoxymethylene composite, where the crystallisation temperature increases due to the nucleating nature of the graphene [32]. Both the commercial PHA and Cutiderm had higher melting peaks compared to our composites, which might hinder the processing capability of both materials as composites for incorporating other materials and also their degradation capability.

### 2.5. XRD

XRD was performed to confirm the presence of the different phases and structures of the material in the composite samples.

**Figure 6:**
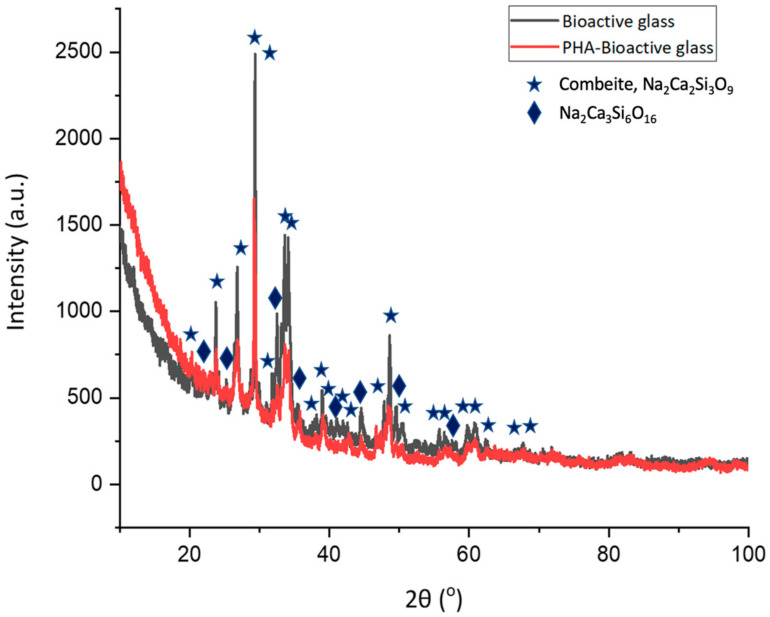
The presence of both crystalline and amorphous phases of bioactive glass can be seen even after the incorporation with the copolymer, but at a lower spectrum intensity as confirmed using XRD.

Figure 6 shows the presence of both crystalline and amorphous phases of bioactive glass with the main crystalline phase combeite, Na_2_Ca_2_Si_3_O_9_ (ICDD 00-001-1078), and sodium calcium silicate, Na_2_Ca_3_Si_6_O_16_ (ICDD 00-001-1067), seen for both tested samples, but also a low spectrum intensity was seen after the incorporation of bioactive glass into the copolymer using facile blending, which suggests the miscibility of both materials [22,33].

Bioactive glass is known to possess semi-crystalline phases due to the presence of sodium that reacts with nitrate groups in the composition, which promotes sodium nitrate crystallisation phases during the sol-gel process. In addition to high working temperatures, which promote crystallisation, the silica and phosphate-rich content in the glass contributes to crystallisation during the ageing and drying processes. The nucleation of new crystalline phases led to obvious XRD diffraction peaks in the main crystalline phase combeite, Na_2_Ca_2_Si_3_O_9_ (ICDD 00-001-1078), and sodium calcium silicate, Na_2_CaSiO_4_ (ICDD 00-001-1067), for both the PHA and PHA-bioactive glass composites seen in Figure 6. Similarly, other studies have shown the presence of these crystalline phases, when sol-gel-derived bioactive glass sintered at high temperatures of 700 °C was used, where it has been reported to be due to the decomposition of sodium nitrate, especially combeite, which appears at several peaks as they influence the bioactivity level of the composite [22,28,34]. A decrement in the diffraction intensity of the composite after the incorporation of bioactive glass was observed, which is mainly due to the blocking of diffraction signals due to the presence of bioactive glass components and also the heterogeneity of both components as the copolymer possesses less pronounced crystalline peaks compared to the bioactive glass, which provides lower values for the composite.

### 2.6. SEM

**Figure 7:**
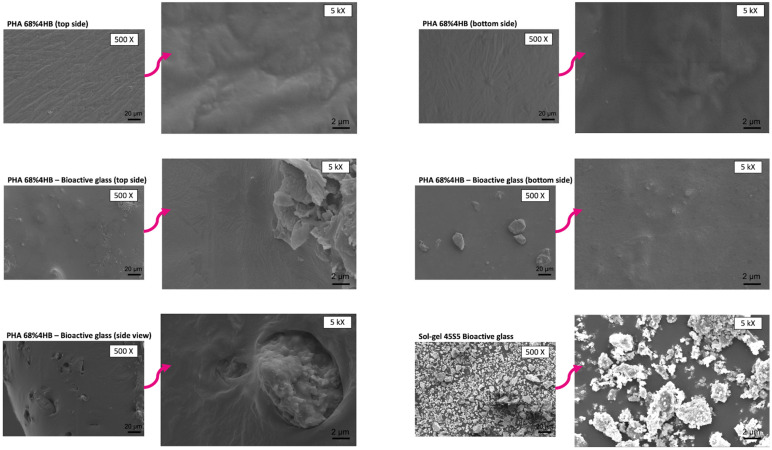
SEM images show a smooth structure but with river line marks in the PHA 68% 4HB-based composites, with the dispersion of bioactive glass powder within.

The morphology of the composites was viewed using SEM, in which the PHA 68% 4HB-based composites in general showed a rather smooth structure with river line marks. There was a rougher surface with the addition of bioactive glass on the inside and on top of the composite itself, indicating most of the bioactive glass powder was dispersed and settled mainly within the copolymer, as seen in Figure 7.

**Figure 8:**
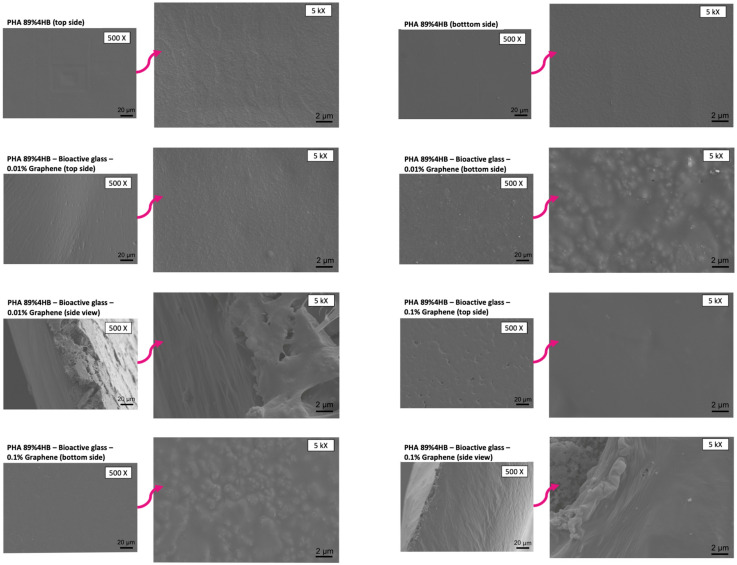
Similar observation for PHA 89% 4HB, with the further incorporation of bioactive glass layer and graphene dispersion throughout the structure, imaged using SEM.

Similar observations were seen for the PHA 89% 4HB-based composites, including those that incorporated bioactive glass and graphene, where smooth river line marks were seen on the composites, as shown in Figure 8. The side view of the composites showed that a bioactive glass layer was formed and attached to the copolymer, with graphene dispersion within the whole structure. It was also evidenced that, as the concentration of graphene increased, the formation of irregular pores and river lines could clearly be observed. This is similar to a study by Vigneswari et al. that showed the formation of irregular pores on the copolymer, suggesting this might be due to the evaporation of the solvent, in this case, the fast evaporation of chloroform during the fabrication of the composite [35]. There is also the potential of artefacts being created on the surface due to the sample preparation, since a previous study by Girvan suggested that the freezing of samples (cryo-SEM) is more likely to produce fewer artefacts before being gold-sputtered, as the technique ensures the retaining of water when viewing the samples under a microscope [36]. The facile blending technique was employed, where each material was individually solubilised before being combined and sonicated to form a smooth suspension before being cast using the solvent casting method. The surface functionalisation of the composite allows more incorporated materials to form on the surface, which leads to a large surface area, which enables a large amount of water to be enclosed, i.e., ensuring the wettability of the composite can be retained. The cross-sections of the composites confirmed the presence of both bioactive glass and graphene surface-functionalised and no obvious segregation was observed within the structure. This is particularly important to ensure that the stable release of ions of each component is in parallel with the gradual decomposition of the composite.

**Figure 9:**
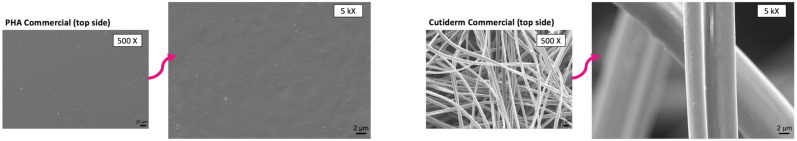
A smooth structure is present in the PHA-based commercial composite and Cutiderm (calcium-alginate-based composite) shows a fibrous-like structure.

Figure 9 shows the morphological structure of the commercial composite, the PHA-based film, which showed a similar smooth structure; meanwhile, a fibrous-like structure can be seen for Cutiderm, which is a calcium-alginate-based membrane.

### 2.7. DMA

**Figure 10:**
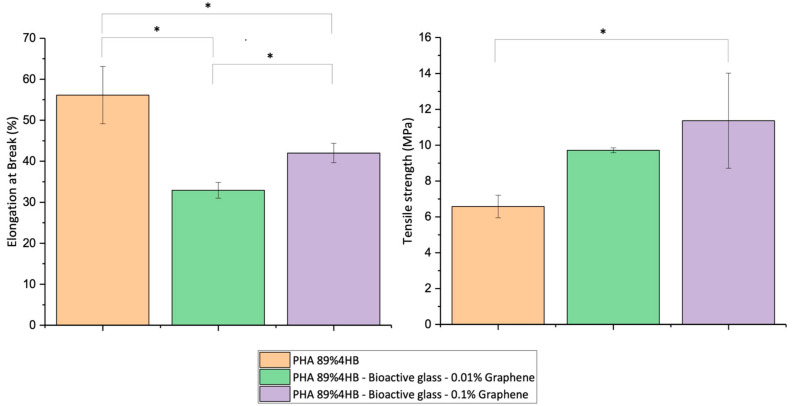
Increment in the mechanical tensile abilities of the composites with an increment in the graphene concentration compared to the pure copolymer, PHA 89% 4HB. Mean values of three replicates accompanied by * indicate a significant difference with a *p*-value < 0.05.

Figure 10 summarises the tensile strength and elongation at break (EAB) characteristics, obtained using the dynamic mechanical analyser (DMA), of the composites. The results show significant differences in the composites for EAB, where the PHA 89% 4HB copolymer recorded the highest value at around 56%, compared to both the PHA 89% 4HB-Bioactive glass—0.01%Graphene and PHA 89% 4HB-Bioactive glass—0.1%Graphene composites, for which the EAB values were compromised.

Interestingly, EAB increased as the percentage of the incorporated graphene increased from 33% with 0.01% graphene to 42% at the concentration of 0.1%. Meanwhile, tensile strength showed a trend of increasing as the graphene percentage increased, compared to the PHA 89% 4HB copolymer, with a significant difference seen in the PHA 89% 4HB copolymer, between 6.5 MPa and 11 MPa, after the incorporation of bioactive glass and 0.1% of graphene.

The mechanical abilities of the composites after the incorporation of both prominent materials, bioactive glass and graphene, showed rather expected trends. The incorporation of inorganic glass, in general, was expected to compromise the mechanical ability of the composite. A decrease in EAB was seen potentially due to the presence of bioactive glass, which exhibits brittle properties. A study by Ding et al. reported that the introduction of 58S bioactive glass content decreased the strength and strain of the PHB/PCL blend, which was most likely due to changes in the crystallinity structure of the composite and the potentially remaining water molecules and residue of bioactive glass precursors that affect the mechanical points of the matrix [13,37,38]. However, interestingly, the EAB increased as the percentage of incorporated graphene increased, which indicates graphene plays a role in enhancing the mechanical flexibility and elasticity of the composite, similar to a study by Sridhar et al., which reported improved mechanical properties with increased storage modulus values as the graphene concentration increased, attributed to the better immobilisation of the polymer chain onto the graphene surface via physical crosslinks in the crystalline regions [39]. Our study showed a composite with a satisfactory range of maximal tensile strength at 11 MPa and EAB at 42% for the production of wound dressings after the incorporation of both bioactive glass and graphene, as, in general, the combination of high tensile strength and modulus with good flexibility is required. A study by Elsner et al. developed a polyglycolide-based composite layer with a maximal stress of 24 MPa and strain of 55%, which was an optimal mechanical combination for a wound dressing compared to most other dressings currently used or studied [20]. Hence, the mechanical range of our copolymer suggested a rather strong and durable composite, which affects the composite’s resorbable rate.

### 2.8. Water Contact Angle

The water contact angle corresponds to the angle formed by the intersection of the liquid–solid interface. Table 3 shows the wettability of the two sides of each composite tested, side A and side B, where surface differences were created when preparing the samples by solvent casting. The PHA 68% 4HB composite, which had a lower percentage of 4HB, had a higher water contact angle compared to the PHA 89% 4HB composite, indicating that the former was more hydrophobic. An even lower water contact angle was seen for the composites after the incorporation of bioactive glass and graphene, suggesting the even higher hydrophilic nature of the composite. However, of note, there was a slight increase in the angle after a higher percentage of graphene was incorporated. Comparatively, the commercial PHA had a low water contact angle as well, and the commercial Cutiderm (not shown here) was unable to capture any angle due to the highly absorbent properties of the composite.

Surface properties are one of the key parameters for any biomaterial to be used biologically, as a moderate hydrophilicity will help to improve cell adhesion and determine its performance in tissue engineering applications. A previous study by Piarali et al., which utilised L929 fibroblast cells with PHA-based meshes activated with Amhelin and Dispersin B, showed the lowest contact angle between the PHA mesh samples, which correlated to the sample with the highest cell density, thus suggesting hydrophilicity helps to improve the interaction between the composite and cell adhesion and growth [19]. Similar to our study, it was mentioned by Vigneswari et al. in their study that the P(3HB-*co*-4HB) copolymer exhibits a high water contact angle, which suggests the hydrophobic surface of the material. However, later in their study, the several weight percentages of P(3HB-*co*-4HB) with the incorporation of collagen showed that the study optimised the collagen concentration up to 15 wt% for a suitable P(3HB-*co*-4HB) molar fraction of 50 mol%, enhancing the hydrophilicity and biocompatibility of the produced scaffold [35]. In our study, we utilised bioactive glass, which is known to be highly hydrophilic [40], for surface functionalisation and modification to improve the wettability of the hydrophobic polymer. Although the incorporation of graphene at a higher concentration into the copolymer showed a slight increase in the water contact angle, our study showed that the appropriate combination of both bioactive glass and graphene, which was confirmed using SEM, resulted in a decreased water contact angle at the interface with the composite. The composites were shown to be enhanced in terms of wettability.

### 2.9. Viability of Cells

**Figure 11:**
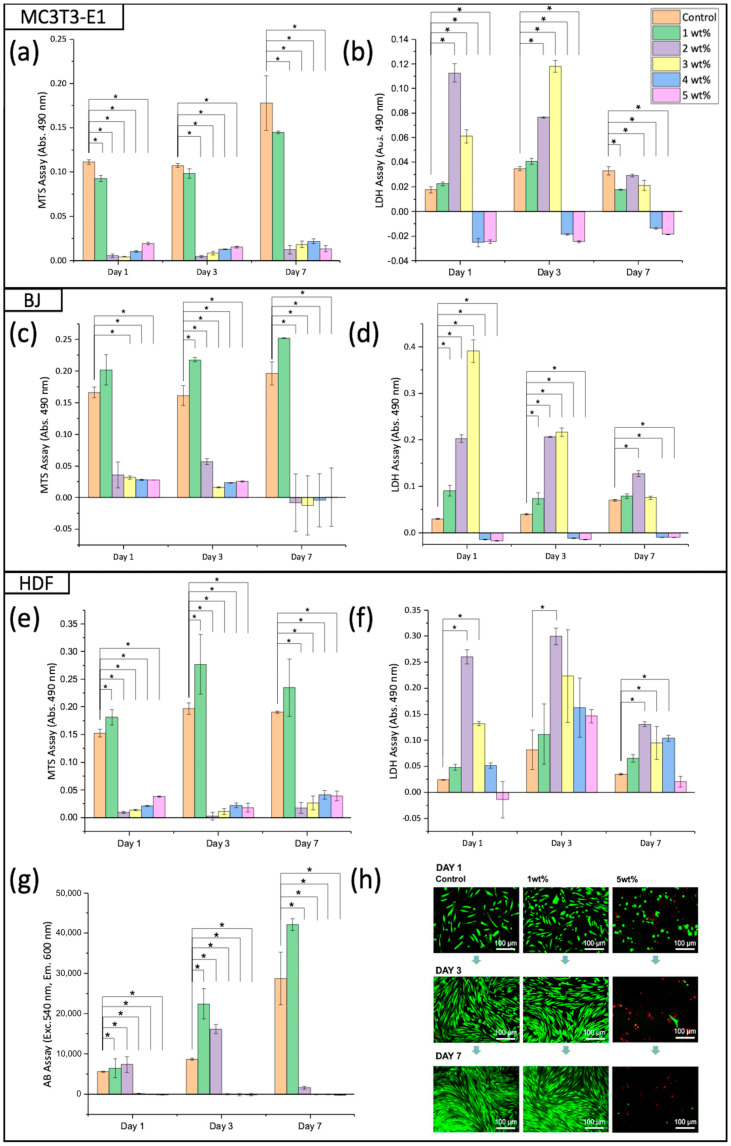
Optimisations of sol-gel-derived bioactive glass dilutions using viability assays against several cells. (**a**) MTS and (**b**) LDH assays against MC3T3 pre-osteoblast cells; (**c**) MTS and (**d**) LDH assays against the BJ human dermal cell line; (**e**) MTS, (**f**) LDH assays, (**g**) Alamar blue (AB), and (**h**) live–dead assays against HDF primary cells. Mean values of three replicates accompanied by * indicate significant differences with a *p*-value < 0.05.

The sol-gel-derived bioactive glass was characterised in cell culture to obtain its best concentration using several biological assays, as shown in Figure 11. The study was performed using three types of cells, firstly with the pre-osteoblast cell line MC3T3-E1 derived from mice, shown in Figure 11a,b for MTS and LDH, respectively. The MTS measurement of the metabolic activity of cells showed that the MC3T3-E1 cells favoured lower concentrations of bioactive glass, and the LDH release by the cells also showed lower cytotoxicity levels at lower concentrations of bioactive glass. The dilutions were then tested against the BJ human dermal fibroblast cell line, where the trends of both the metabolic activity and cell viability measured using MTS and LDH, shown in Figure 11c,d, respectively, were similar to those seen in the MC3T3-E1 cells. Finally, the human dermal fibroblast (HDF) primary cells were used as a representative of native skin to investigate the copolymer and composite potential in wound healing applications with the potentiality of non-load-bearing hard tissue regeneration against pre-osteoblast cells.. Figure 11e,f show the MTS and LDH assays, respectively; similar results were obtained to those for the MC3T3-E1 cells and BJ cells. The more sensitive fluorimetric AB assay, with additional cell viability, was visualised using the live–dead assay against HDFs, as shown in Figure 11g,h. These were conducted as thorough confirmation indicators, which showed optimisation at low concentrations of bioactive glass dilutions at 1 wt%, which supports higher cell viability, compared to TCP and other higher concentrations of bioactive glass dilutions.

A similar trend was seen where the cells had the highest metabolic activity with the lowest concentration of bioactive glass at 1 wt%, compared to other higher concentrations. The LDH released by cells increased as the concentration of bioactive glass increased, indicating increased cytotoxicity. A similar study reported the importance of controlling the quantity of bioactive glass, where at high concentrations, a reduction in VEGF secretion was seen, mainly due to the cytotoxicity induced by the material [41]. Another previous study that used the same bioactive glass components but with a different method of derivation also showed similar results, where higher doses of the material led to cytotoxic effects towards dental pulp stem cells [42]. Keshaw et al. reported that the optimal concentration of bioactive glass they used was in the range of 0.01–0.1 wt%, whereas in our study, we only examined a rather higher concentration at 1 wt%. Keshaw et al. did not mention the synthesising route of their bioactive glass; however, the original melt-derived route for 45S5 bioactive glass has been reported to induce lower bioactivity levels and lower resorbable rates compared to sol-gel-derived bioactive glass [27]. The cells in the presence of bioactive glass were monitored and tested at several time points, on days 1, 3, and 7. The initial positive response of these cells towards bioactive-glass-optimised dilutions, specifically within 24 h, is particularly important as bioactive glass plays an important role in modulating anti-inflammatory and growth factors upon the dissolution of its ions as an immediate effect during initial wound covering, as this continues by promoting vascularisation for an efficient process of wound healing. A longer context, i.e., for a leaving-on wound dressing, which is used and replaced every 5 days, also requires a similar continuous positive response from the materials, and these were shown using cells sustaining their high metabolic activity and low level of cytotoxicity for an extended time point until day 7. Similar observations of MTS and LDH were also seen for AB and live–dead assays, where the HDF cells showed a favourable response towards low concentrations of bioactive glass dilutions. It was both quantitative and qualitative, respectively, using these two assays. Taken together, all our composites were optimised at 1 wt% of bioactive glass content, which showed optimised mechanical and physical properties, including enhanced wettability shown using the water contact angle method. The optimisations of bioactive glass ensured an optimal growth environment for cells, which could contribute to the secretion of angiogenic growth factors that enhance the proliferation of microvascular endothelial cells and subsequent revascularisation, which is an important factor in accelerating wound management.

## 3. Conclusions

In conclusion, P(3HB-*co*-4HB) copolymers were produced using the fermentation method in shake flasks and in a bioreactor, although a low biomass yield was obtained with both systems in this study, especially with the latter fermentation system. It somehow also affected the 4HB monomer composition, which was compared between two batches using shake flask fermentation, which highlights the importance of regulating strain conditions. The plasmid maintenance of the strain highly depends on not only internal functionalisation but also external factors, which directly affect the biosynthesis of the intracellular copolymer.

Changes in the amount of 4HB monomers in the copolymer can increase the processing window, and mechanical properties and subsequently porous and laminar composite structures can be created by the incorporation of bioactive glass and graphene. The functionalisation of these materials also enhances the wettability of the composite, which could potentially facilitate and improve cell adhesion.

Taken together, the surface-functionalised composite formulated in this study has the potential to be utilised as a therapeutic dressing for topical applications, as well as for other applications, including drug encapsulation and release, patches for skin lesions in anti-cancer treatment, and non-load-bearing healing and regenerative medicine.

## 4. Materials and Methods

### 4.1. Materials

*C. malaysiensis* USM1020-harbouring plasmid pBB2-PC_1020_, a derivative of pBBR1MC-2 with a copy of the phaC gene from the same strain (Universiti Sains Malaysia) [9], was grown on nutrient agar (NA) or in nutrient broth (NR) containing 50 ug/mL of kanamycin (all materials for NA and NR were obtained from Merck Millipore (Sigma Aldrich, Poole, UK) and Oxoid (Thermo Fisher Scientific, Loughborough, UK), respectively). We also employed the following: KH_2_PO_4_ (Sigma Aldrich, Poole, UK), K_2_HPO_4_ (Sigma Aldrich, Poole, UK), (NH_4_)_2_SO_4_ (Thermo Fisher Scientific, Loughborough, UK), MgSO_4_·7H_2_O (Thermo Fisher Scientific, Loughborough, UK), kanamycin sulphate (VWR chemicals, Lutterworth, UK), trace element solution (TE) (FeSO_4_·7H_2_O, MnCl_2_·4H_2_0, CoSO_4_·7H_2_O, CaCl_2_·2H_2_O, CuCl_2_·2H_2_O, ZnSO_4_·7H_2_O, HCl) (all materials for the TE solutions were purchased from Sigma Aldrich, Poole, UK, unless otherwise stated), glycerol (Thermo Fisher Scientific, Loughborough, UK), 1,6-hexanediol (Sigma Aldrich, Poole, UK), 1,4-butanediol (Sigma Aldrich, Poole, UK), chloroform (VWR chemicals, Lutterworth, UK), methanol (Thermo Fisher Scientific, Loughborough, UK), fluorescein isothiocyanate fluorescent dye (Sigma Aldrich, Poole, UK), nitric acid (Thermo Fisher Scientific, Loughborough, UK), tetraethyl orthosilicate (Sigma Aldrich, Poole, UK), triethyl phosphate (VWR chemicals, Lutterworth, UK), sodium nitrate (VWR chemicals, Lutterworth, UK), calcium nitrate tetrahydrate (Sigma Aldrich, Poole, UK), graphite flakes (332461, Sigma Aldrich, Poole, UK), CellTiter 96 Aqueous One Solution Cell Proliferation Assay (MTS) and CytoTox-ONE Homogenous Membrane Integrity Assay (LDH) (both were purchased from Promega (Southampton, UK)), Live/Dead™ Viability/Cytotoxicity Kit for mammalian cells (Thermo Fisher Scientific, Loughborough, UK), trypsin-EDTA (Sigma Aldrich, Poole, UK), high-glucose DMEM (Thermo Fisher Scientific, Loughborough, UK), MEM α (nucleosides, no ascorbic acid) (Thermo Fisher Scientific, Loughborough, UK), Eagle’s Minimum Essential Medium (EMEM) (ATCC, Middlesex, UK), foetal bovine serum (Thermo Fisher Scientific, Loughborough, UK), penicillin–streptomycin (Sigma Aldrich, Poole, UK), and trypan blue solution (Sigma Aldrich, Poole, UK). Adult human dermal fibroblasts (HDFa) (Thermo Fisher Scientific, Loughborough, UK), MC3T3-E1 cell line from mice (Sigma Aldrich, Poole, UK), BJ fibroblast cell line, human (ATCC, Middlesex, UK), Gibco Trypan Blue solution (0.4%) (Thermo Fisher Scientific, Loughborough, UK), Polyhydroxybutyrate/Polyhdroxyvalerate 8% (PHB92/PHV8, BV301025) film (Goodfellow Cambridge Limited, Huntingdon, UK), and calcium alginate wound dressing (Cutiderm) (JFA Medical Ltd., Ashton-under-Lyne, UK) were also used.

### 4.2. Methods

#### 4.2.1. Preparation of the Inoculum

P(3HB-*co*-4HB) was recovered and synthesised from *C. malaysiensis* USM1020-harbouring plasmid pBB2-PC_1020_, a derivative of pBBR1MC-2 with a copy of the phaC gene from the same strain (Universiti Sains Malaysia), as previously reported [6,9]. Briefly, the strain was kept in a glycerol/NB stock containing 50 μg/mL kanamycin at −80 °C for long-term storage. The strain was cultured on NA with 50 μg/mL of kanamycin for plasmid maintenance and subsequently incubated for 48 h at 30 °C. It was then precultured in NB (containing 50 μg/mL of kanamycin) and incubated using shaking at 30 °C with an agitation speed of 200 rpm (Incu-Shake FL18-750R, Sciquip, Newtown, UK) for 12 h.

For the inoculum preparation, 0.1 g/L of the total culture from the pre-culture was transferred into a sterilised mineral salt medium (MSM) consisting of 3.70 g/L KH_2_PO_4_, 5.80 g/L K_2_HPO_4_, 1.1 g/L (NH_4_)_2_SO_4_, 0.2 g/L MgSO_4_·7H_2_O, 50 μg/mL kanamycin, and 1.0 mL/L trace element (TE) solution (2.78 g FeSO_4_·7H_2_O, 1.98 g MnCl_2_·4H_2_0, 2.81 g CoSO_4_·7H_2_O, 1.67 g CaCl_2_·2H_2_O, 0.17 g CuCl_2_·2H_2_O, and 0.29 g ZnSO_4_·7H_2_O per L of 0.1 mol/L HCl). The medium was then supplemented with a mixture of 2 carbon sources, 1,6-hexanediol and 1,4-butanediol, at a ratio of 1:5 with a total concentration of 0.75% (*w*/*v*) carbon.

#### 4.2.2. Biosynthesis of the P(3HB-*co*-4HB) Copolymer

Two methods of culturing the strain were employed for the production of the P(3HB-*co*-4HB) copolymer- the shake flask, and the bioreactor.

##### Fermentation in the Shake Flask

For the shake flask fermentation, inocula (see Section 4.2.1) were transferred into 250 mL Erlenmeyer flasks containing 50 mL of sterilised MSM. The fermentation was carried out in triplicate at 30 °C with agitation at 200 rpm for 48 h.

The optical density of the culture was measured every 24 h to monitor the growth profile of the culture at a wavelength of 540 nm (Jenway 6300 spectrophotometer, Cole-Parmer, Eaton Socon, UK).

##### Fermentation in the Bioreactor

For the fermentation in the bioreactor, the inocula (see Section 4.2.1) were transferred to a STR bioreactor (Biostat Sartorius Cplus, Sartorius, Göttingen, Germany) containing 20 L of sterilised MSM. The experiment was carried out in triplicate at 30 °C with an agitation speed of 250 rpm and aeration rate of 1 vvm for 48 h.

The optical density of biomass was monitored every 24 h to obtain the growth profile of the culture at a wavelength of 540 nm (Jenway 6300 spectrophotometer, Cole-Parmer, Eaton Socon, UK).

#### 4.2.3. Extraction and Recovery of the P(3HB-*co*-4HB) Copolymer

The intracellular P(3HB-*co*-4HB) copolymer was recovered via extraction with chloroform (1 g lyophilised cell: 200 mL chloroform) for 48 h. The mixture was then filtered (Whatman qualitative filter paper, Grade 1, circles with a diam. of 150 mm, Sigma Aldrich, Poole, UK) and concentrated via evaporation at 60 °C using a rotary evaporator (Stuart BIBBY RE200, Bibby Scientific, Loughborough, UK). The pure crude copolymer was precipitated in chilled methanol (−20 °C), filtered (0.45 μm PTFE filter membrane, Thermo Fisher Scientific, Loughborough, UK), and left to dry at room temperature (~19 °C).

#### 4.2.4. Endotoxin Removal from the Copolymer

Endotoxin removal was conducted by dissolving 2 g of the copolymer firstly in 100 mL chloroform (2% *w*/*v*) at 30–40 °C. Subsequently, 3 aliquots of 110 μL/g of H_2_O_2_ were added to the dissolved copolymer at 20 min intervals. The dissolved copolymer was then left to cool down at room temperature (~19 °C) and chloroform was allowed to evaporate. It was then precipitated in chilled methanol, left stirring overnight, and filtered (0.45 μm PTFE filter membrane, Thermo Fisher Scientific, Loughborough, UK) before being left to dry at room temperature (~19 °C) for 4 days before any further characterisations.

#### 4.2.5. Proton Nuclear Magnetic Resonance (^1^H-NMR) Spectroscopy

^1^H-NMR was employed to study the crude copolymer identity, structure, and monomer composition. A total of 10 mg of the purified P(3HB-*co*-4HB) copolymer and commercial PHB92/PHV8 were stirred in deuterated chloroform (CDCl_3_) at room temperature (~19 °C) for 15 min. Cutiderm, a calcium-alginate-based commercial wound dressing, was dissolved in a solution containing sodium carbonate in heavy water (D_2_O) for 15 min at room temperature (~19 °C). Different solvents were used according to the dissolving nature of each material and according to the guidelines for running ^1^H-NMR. Tetramethylsilane (TMS) was used as the internal standard (δ_TMS_ = 0 ppm) and all samples were then subjected to 400 MHz ^1^H-NMR analysis (Bruker Avance Neo 700, Bruker, Cambridge, UK).

#### 4.2.6. Preparation of the Sol-Gel-Derived Bioactive Glass

The sol-gel-derived 45S5 bioactive glass was synthesised by following a previously reported method with slight modifications [34]. Briefly, 33.5 mL of TEOS were added to 50 mL of 0.268 M of nitric acid for 1 h at room temperature (~19 °C). Subsequently, 2.9 mL of TEP, 20.64 g of Ca(NO_3_)_2_·4H_2_O, and 13.43 g of NaNO_3_ were added at 45 min intervals between each, allowing each material to fully dissolve. The solution was left stirring overnight at room temperature (~19 °C). The gelation process was initiated by sealing and placing the mixture in an oven at 35 °C for 3 days, aged for another 2 days at 60 °C, before subsequent drying at 110 °C for 2 days. Finally, the dried gels were sintered inside a furnace (Carbolite RHF 15/3 furnace, Carbolite Gero, Hope Valley, UK) for 1 h at 700 °C at a rate of 5 °C/min. The powder was then ground and sieved at 70 μm for further casting and characterisation.

#### 4.2.7. Preparation of the Liquid Exfoliated Graphene

A graphene suspension was obtained via the liquid exfoliation of graphite in chloroform. Graphene suspension concentrations of 0.01 wt% and 0.1 wt% were chosen based on the literature [43]. Graphite flakes were firstly bath-sonicated (Fisherbrand, FB 15047, Heated Ultrasonic Bath, Thermo Fisher Scientific, Loughborough, UK) in 10 mL of chloroform at 37 kHz for 7 h and centrifuged at 3500 rpm for 30 min to obtain a homogenous and monolayer formation of graphene layers within the suspension for further casting and characterisation.

#### 4.2.8. Casting of the P(3HB-*co*-4HB) Composite

The P(3HB-*co*-4HB) composite was cast using an adapted solvent casting protocol, whereby the crude copolymer was dissolved in chloroform at 5 *w*/*v*% [35]. A facile blending technique was employed for the fabrication of the composite, where sol-gel-derived 45S5 bioactive glass powder (1 and 5 wt%) and liquid exfoliated graphene (0.01 and 0.1 wt%) were individually dissolved in chloroform before being subjected to bath sonication (Fisherbrand, FB 15047, Heated Ultrasonic Bath, Thermo Fisher Scientific, Loughborough, UK) at 37 kHz for 4 h to obtain a homogenously dispersed solution. Ice was added to the bath sonicator every 30 min. The solution was then cast in 6 cm diameter glass Petri dishes and allowed to evaporate in a fume hood for 96 h, resulting in a free-standing composite. The composite thickness was measured with a thickness gauge (Fowler Ultra Cal V Electronic Calipers, IP67, Fowler, Cole Palmer, St. Neots, UK).

### 4.3. Characterisation Techniques

#### 4.3.1. Attenuated Total Reflectance—Fourier-Transform Infrared (ATR-FTIR)

ATR-FTIR (Perkin Elmer Instruments, Beaconsfield, UK) spectroscopy was carried out to analyse the presence of functional groups and changes in the interphase layer between each component in the biocomposite. The composite samples were cut into 5 mm squares of 2 mm thickness and positioned firmly on the small diamond crystal area. The spectrum was recorded in the absorbance range of 400–4000 cm^−1^, at 4 cm^−1^ resolution, for ten scans at room temperature (~19 °C).

#### 4.3.2. Differential Scanning Calorimetry (DSC)

DSC was employed to obtain the thermal profile of the samples including the glass transition temperature (T_g_), melting temperature (T_m_), and crystallisation temperature (T_c_). Thermograms were generated using a DSC25 instrument (TA-Instruments, New Castle, DE, USA) by placing 8 mg of each sample, contained within a Tzero lid-sealed aluminium pan, and analysing accordingly with a heating profile ranging from −50 to 300 °C, at a rate of 10 °C/min for 35 min, excluding pre-heating and post-heating times.

#### 4.3.3. Scanning Electron Microscopy (SEM)

The morphological structure of the composites was viewed under SEM (Zeiss EVO HD, Jena, Germany). Triplicates of each sample group were cut into ~5 mm squares, mounted on stubs, and coated with 95% gold and 5% palladium (Polaron E5000 Sputter Coater, Quorum Technologies, Laughton, UK). The images of each sample were viewed at the central position of magnifications 500× and 5000×; the fibre diameter was measured and captured using the Zeiss EVO HD in-built software (i.e. Zen 2.5, version 1).

#### 4.3.4. X-ray Diffractometry (XRD)

XRD was employed to detect the presence of crystalline phases in the sol-gel-derived bioactive glass in the P(3HB-*co*-4HB) composite. The bioactive glass was ground and pressed; meanwhile, the composite was mounted on a flat plate geometry. The scattering of the X-ray beam diffraction was recorded as XRD spectra using a Bruker D8 advance diffractometer (Bruker, Coventry, UK) equipped with Ni-filtered Cu Ka radiation. Data were collected using a Lynx eye detector with an incident slit of 0.2 mm and step size of 0.019° over an angular range 2θ from 10 to 100°.

#### 4.3.5. Dynamic Mechanical Analysis (DMA)

The tensile test was carried out via Dynamic Mechanical Analysis (DMA, Discovery DMA 850—TA Instruments, New Castle, UK) at room temperature (~19 °C). The test was performed to study the tensile strength (TS), Young’s Modulus (YM), and elongation at break (EAB) of the samples. Three discrete sections of each composite sample were prepared and cut in a uniform rectangle shape (2 cm length, 0.5 cm width, and 0.2 cm thickness). Each sample was gripped at both sides with ridged tensile clamps and slowly exerted horizontally at a crosshead speed of 0.5 mm/min until failure. The stress–strain curves obtained were assessed in terms of tensile strength (TS), elongation at break (EAB), and Young’s modulus (E). The results are presented as mean values ± SD.

#### 4.3.6. Water Contact Angle

The wettability nature of the samples was assessed via water contact angle measurements (CAM 200 Optical Contact Angle Meter instrument, KSV, Espoo, Finland). The surface–liquid interface was established by measuring the placement of a liquid (water) drop at the centre of the sample, with a circular algorithm technique. The angle was then captured at both sides of each sample, orientated as side A and side B, and measured using the CAM 200 analysis software, version 3.922.

#### 4.3.7. Cell Metabolic Assays

The sol-gel-derived bioactive glass was individually assessed using its dilutions to optimise the concentration for the composite using several biological assays as outlined below.

Human dermal fibroblasts (adult primary cells), BJ cell line, and the mouse osteoblastic MC3T3-E1 cell line were cultured in DMEM with high glucose, EMEM, and MEM α (nucleosides, no ascorbic acid), respectively, supplemented with 10% foetal bovine serum and 1% of penicillin/streptomycin. All cells were subcultured using a standard trypsinisation protocol (1% *v*/*v* trypsin-EDTA) every 3 days under standard humidified cell culture conditions (37 °C and 5% CO_2_).

The optimisation of the concentrations of bioactive glass was assessed by firstly preparing dilutions at several concentrations (0.5, 1, 1.5, 2, 3, 4, and 5 *w*/*v%*), stirred in the culture media, respective to each cell, for 24 h to allow maximum ion dissolution and filtered (0.2 μm PES syringe filter, Thermo Fisher Scientific, UK) to sterilise the dilutions. Once the cells reached 80% confluency as viewed using a microscope (Olympus CK2 Inverted Phase Contrast Microscope, Microscopy Technologies, Evident Corporation, Tokyo, Japan), the cells were trypsinised using the standard protocol, counted using viable cell staining with the trypan blue dye exclusion method and haemocytometer, seeded at a density of 7000 cells/well in 150 μL of complete growth medium in 96-well tissue culture plates (Thermo Fisher, Loughborough, UK), and incubated accordingly (37 °C and 5% CO_2_). Each dilution concentration had 3 replicates, including tissue culture plastic (TCP) used as a control.

##### Metabolic Activity Using MTS and AB

The metabolic activity of the cells was assessed and compared using two indicators that were reduced by living cells. The seeded cells were first analysed using the fluorimetric Alamar blue (AB) assay followed by the colorimetric MTS assay.

The AB assay was used to study the metabolic activity through the proliferation of cells on days 1, 3, and 7, when 10 *v*/*v*% of the AB dilutions was added into each well and incubated for 4 h. Fluorescence values were measured at an excitation wavelength of 540 nm and emission wavelength of 600 nm using a BioTek FLx800 plate reader (BioTek, Swindon, UK).

The comparative metabolic activity of cells was assessed using MTS to study the possibility of toxicity induced by bioactive glass concentrations and its effect on the cells’ proliferation and viability on days 1, 3, and 7. A total of 20 μL of the CellTiter One reagent was added into each well and incubated at 37 °C with 5% CO_2_ for 3 h. Subsequently, the incubated media from each well were transferred into a new 96-well plate, with background control of sample containing media only, and the absorbance was read at a wavelength of 490 nm using a Tecan M200 microplate reader (Tecan).

##### LDH Assay

The LDH assay was employed to further study the cell cytotoxicity (cell membrane leakage) of the seeded cells in response to the bioactive glass dilutions on days 1, 3, and 7. A total of 50 μL of CytoTox-One reagent was added to 50 μL of cultured media in a 96-well plate. The plate was wrapped using aluminium foil and incubated at room temperature (~19 °C) for 30 min. Subsequently, 50 μL of the stop solution was added into each well and the samples, including the lysed control, which was made by prior treatment with Triton X-100, were read at a wavelength of 490 nm using a Tecan M200 microplate reader (Tecan).

##### Live and Dead Cell Imaging

Live and dead cell imaging was employed to observe the viability of the seeded cells at the several concentrations of bioactive glass on days 1, 3, and 7. The samples were firstly washed in phosphate buffer solution (PBS), before 2 μM Calcein AM and 4 μM EthD-1 (Live/Dead viability/cytotoxicity kit for mammalian cells, Thermo Fisher Scientific Inc.) were subsequently added; samples were thereafter wrapped in foil and incubated for 30 min at room temperature (~19 °C) prior to viewing under a fluorescence microscope (Leica Microsystems, Milton Keynes, UK) and a QImaging QICam Mono camera (Media Cybernetics, Slough, UK). The QCapture software, version 11.0.2, Media Cybernetics, Slough, UK) was used to take the images, which were analysed using ImageJ version 1.54g (available to download at https://imagej.net/ij/download.html, accessed on 8 January 2024).

### 4.4. Statistical Analysis

All results presented are expressed as the mean values ± SD of the number of replicates stated in each characterisation section. All the data were statistically analysed using the OriginLab version 2019 (9.60) software. The significance difference was determined using a one-way ANOVA with a *p*-value < 0.05 being considered significant (*).

## Figures and Tables

**Table 1:** Percentage of the crude copolymer yielded using shake flask fermentation and inoculation in a bioreactor.

Method of Inoculation	Optical Density Measurements to Monitor Bacterial Growth (A), by Trials					Initial Weight of Lyophilised Cells before Extraction ^1^ (g)	Final Weight of Extracted/Recovered Crude Copolymer ^2^ (g)
		1 (Mean)	2 (Mean)	3 (Mean)	Mean of Three Trials ± SD		
Shake flask	48 h	0.667	0.649	0.625	0.647 ± 0.021	1.25	0.65 (51.72%)
Bioreactor	48 h	0.7	0.682	0.687	0.690 ± 0.019	1.25	0.69 (55.19%)

^1^ Extraction was carried out at a ratio of 1 g of lyophilised cells: 200 mL of chloroform. ^2^ Recovery was carried out at a ratio of 1 g of lyophilised cells: 150 mL of chilled methanol.

**Table 2:** ^1^H-NMR results of the P(3HB-*co*-4HB) copolymer produced using shake flask fermentation showed a higher ability of 4HB accumulation for batch A, which correlates to the phaCAB operon (with additional copies of the phaC gene) retained in the strain’s plasmid.

Samples (48 h Harvesting Timepoint)	Monomer Composition (3HB:4HB), via ^1^H-NMR
P(3HB-*co*-4HB): batch A	16%:84%
	11%:89%
P(3HB-*co*-4HB): batch B	32%:68%
	31%:69%

**Table 3:** Water contact angle that shows an enhancement in the hydrophilic nature of the PHA-based composites after the incorporation of both bioactive glass and graphene.

Samples	Contact Angle of Water, θ (°)	
	Side A	Side B
PHA 68% 4HB	106.2	103.1
PHA 89% 4HB	87.1	79.5
PHA 89% 4HB—Bioactive glass—0.01% Graphene	46.3	60.6
PHA 89% 4HB—Bioactive glass—0.1% Graphene	57.3	64.6
Commercial PHA	76.9	80.1

## Data Availability

All data and materials are available upon request from the corresponding author. The data are not publicly available due to ongoing research using a part of the data.
